# Distribution of arsenic, silver, cadmium, lead and other trace elements in water, sediment and macrophytes in the Kenyan part of Lake Victoria: spatial, temporal and bioindicative aspects

**DOI:** 10.1007/s11356-019-06525-9

**Published:** 2019-11-20

**Authors:** James Omondi Outa, Chrispin O. Kowenje, Christof Plessl, Franz Jirsa

**Affiliations:** 1 Department of Limnology and Bio-Oceanography, University of Vienna, Althanstrasse 14, 1090 Vienna, Austria; 2 Institute of Inorganic Chemistry, University of Vienna, Althanstrasse 14, 1090 Vienna, Austria; 3 Department of Chemistry, Maseno University, P.O. Box 333, Maseno, 40105 Kenya; 4 Department of Zoology, University of Johannesburg, P.O. Box 524, Auckland Park, 2006 South Africa

**Keywords:** Heavy metals, Winam Gulf, Pollution, *Ceratophyllum demersum*, *Vossia cuspidata*

## Abstract

This is the first detailed report on the distribution of Ni, As, Sr and Ag in water, sediments and macrophytes from Lake Victoria, complemented with recent data on the heavy metals Cr, Cu, Zn, Cd and Pb. The study was conducted over an 11-month period at five sites in the Kenyan part of Lake Victoria: four sites in the Winam Gulf influenced by various anthropogenic pressures including a site near Kisumu City, and one in the main lake, perceived to have lesser direct anthropogenic influence. Compared with the main lake site, the water in Winam Gulf had significantly higher values for electrical conductivity and concentrations of dissolved components: organic carbon and bound nitrogen, as well as major and most trace elements. This contamination is also evident in surface sediments, which contained significantly higher levels of Cr, Zn, As, Ag, Cd and Pb compared with the main lake site. The mean levels of Cr, Cu, Zn, Ag and Pb exceeded probable effect levels at least at one of the gulf sites. The sediments at the Kisumu City site were classified as severely polluted with Cu (up to 259 mg/kg dw) and Pb (up to 1188 mg/kg dw). The sediment cores showed significantly higher levels of Cu, Zn, Ag, Cd and Pb in the surface (0–3 cm) versus subsurface (22–25 cm) layer at the Kisumu City site, indicating increasing pollution by these elements within the last 15 years. This is also the first report on trace elements in the emergent water plant *Vossia cuspidata* and submerged plant *Ceratophyllum demersum* from this lake. Even though the accumulation of most elements is comparable between *C. demersum* (whole plant) and *V. cuspidata* roots, the latter shows a better bioindicative potential. Contamination of the gulf with Ni, Cu, Zn, Ag, Cd and Pb is well mirrored in *V. cuspidata* roots. *V. cuspidata* strongly restricts the acropetal transport of trace elements, and hence using the shoots as fodder does not pose a risk to livestock.

**Electronic supplementary material:**

The online version of this article (10.1007/s11356-019-06525-9) contains supplementary material, which is available to authorized users.

## Introduction

Inland waterbodies are subject to pollution pressure worldwide (Akwiri et al. 2016). The pollutants may be of organic or inorganic nature and have a multitude of origins (Burton 2002). One major group of pollutants is trace elements including heavy metals and metalloids, which pose a threat to wildlife and humans at elevated concentrations (Miller et al. 2004). Although their natural occurrence in waterbodies is highly dependent on the geological background of the catchment area, anthropogenic activities have multiplied the concentrations of some of these elements during the last decades, in particular lead, mercury and copper (Lau et al. 1998; Aras et al. 2012). According to the Blacksmith Institute and Green Cross report by McCartor and Becker (2010), four out of the six top contaminants in the world are heavy metals (Cr, Hg and Pb) and the metalloid (As), from which an estimated 50 million people globally are at risk of toxic pollution at levels above international health standards.

Trace elements show different partitioning in the various compartments of waterbodies according to the physico-chemical properties of the trace elements as well as the properties of the respective compartment (Zhang et al. 2014). The water phase, with its dissolved components, and the sediment are interconnected by biological and chemical processes. Investigating dissolved elements allows only a snapshot at the time of sampling because only a small portion of free metal ions remain dissolved in water (Hou et al. 2013). The other compartments (sediment and biota), however, can act as reservoirs for contaminants through bioaccumulation and trophic transfer (Burton 2002), enabling a more comprehensive assessment (Alonso et al. 2013). Sediments can also act as a source of trace elements in the water column (Galal and Shehata 2015). The elements in sediment are present in bound and ion-exchangeable forms and can adversely affect sediment-dwelling organisms through direct toxicity (Hou et al. 2013). This calls for regularly monitoring the levels of trace elements in sediments. Although aquatic plants are common and easy to identify compared with other biota, the data on their accumulation of trace elements are rather scarce (Remon et al. 2013). Plants accumulate essential metals such as Mg, Fe, Mn, Zn, Cu, Mo and Ni, and some plants also accumulate elements that have no essential biological function (Brankovic et al. 2015). The mineral nutrition of sessile plants depends closely on the immediate growth medium characteristics, making them suitable bioindicators (Remon et al. 2013).

Inland waters and freshwater biodiversity constitute a valuable natural resource in economic, cultural, aesthetic, scientific and educational terms (Dudgeon et al. 2006). Lake Victoria, the largest lake in Africa, is the most important freshwater resource for the local population (Crul 1995), serving more than 30 million people (Awange and Obera 2007). Like many surface waters, it suffers from anthropogenic pressures. Muli (2006) noted that industrial, agricultural and domestic waste discharges have increased the levels of trace elements in the lake. Njuru and Hecky (2005) reported notable inshore-offshore pollution gradients in several large embayments such as Winam Gulf, Murchison Bay and Mwanza Gulf, which are potential recipients of municipal and industrial wastes from rivers and the adjacent cities. Winam Gulf, also called Nyanza or Kavirondo Gulf, is semi-isolated from the main lake, receives input from the larger rivers (about 10% of the total inflow into the lake) and faces high urban pollution from Kisumu City (Njuru et al. 2013). This gulf is therefore of great interest with regard to pollution studies.

Various studies have investigated the heavy metal levels in Lake Victoria. Onyari and Wandiga (1989), Mwamburi and Oloo (1996) and Kishe-Machumu and Machiwa (2003) focused on the distribution of selected trace elements (Cr, Mn, Fe, Cu, Zn, Cd, Hg and Pb) in surface sediments (< 5 cm). Nonetheless, the information on the distribution and bioaccumulation of other trace elements such as Ni, As, Sr and Ag remains very scarce, with only one published study (Ochieng et al. 2006) on Ni and Ag in surface sediments. Ag, for instance, is fast becoming an element of interest in aquatic pollution studies due to its toxicity and increased usage in various consumer products (Plessl et al. 2018; Yuan et al. 2018). Moreover, the distribution of trace elements in sediment cores has received very little attention in Lake Victoria. Ramlal et al.’s (2003) study on sediment cores in the Ugandan part of Lake Victoria focused solely on Hg. The vertical distribution of elements in sediments is important because it reveals the history of pollution, especially in connection with terrestrial anthropogenic pressures (Skwierawski and Sidoruk 2014). Although aquatic plants are abundant in Lake Victoria, comprehensive data on their trace element contents are scarce. Mahommed and Makundi (2002) reported on metals (Mn, Fe, Co, Ni, Zn and Pb) in the water hyacinth *Eichhornia crassipes* (Mart.) Solms in parts of Mwanza Gulf (Tanzania), where plants subject to industrial and domestic wastewater discharges had higher levels of trace elements than those in non-polluted areas. In assessing the contamination of wetland soils and plants around Lake Victoria (Uganda), Nabulo et al. (2008) found that the Benghal dayflower *Commelina benghalensis* L. and *E. crassipes* accumulate Ni, Cu, Zn, Cd and Pb in their tissues. No information is available on the bioindicative aspects of the submerged non-rooted macrophyte hornwort *Ceratophyllum demersum* L. and the abundant shoreline emergent macrophyte hippo grass *Vossia cuspidata* (Roxb.) Griff in Lake Victoria. Studies from Europe (Stanković et al. 2000) and China (Xing et al. 2013) show that *C. demersum* is a good indicator of freshwater pollution by trace elements, while Galal et al. (2017) noted that *V. cuspidata* has a good bioaccumulation ability for Cr, Mn, Co, Ni, Cu, Zn, Cd and Pb in Ismailia Canal, Egypt. We found no other studies on the bioaccumulation of As, Sr and Ag in *V. cuspidata*; this study therefore gives the first insights. Like most grasses, *V. cuspidata* is widely used as fodder for livestock by most communities around Lake Victoria (Sayer et al. 2018). This calls for investigating the potential risk associated with its utilization as animal feed.

This study investigates the general physico-chemical parameters, major and trace elements in water, sediments and two macrophyte species from the Kenyan part of the lake over a period of 11 months from five different sampling areas. The results help to demonstrate the spatial variations in chemical composition as well as potential temporal changes in the water phase and the sediments. The analyses of sediment cores from those five different sites provide a first insight into the history of trace element deposition. The trace elements in the two macrophytes are a first step in determining their bioindicative abilities and the possible risk posed by using *V. cuspidata* as fodder.

## Materials and methods

### Study area descriptions

**Fig. 1:**
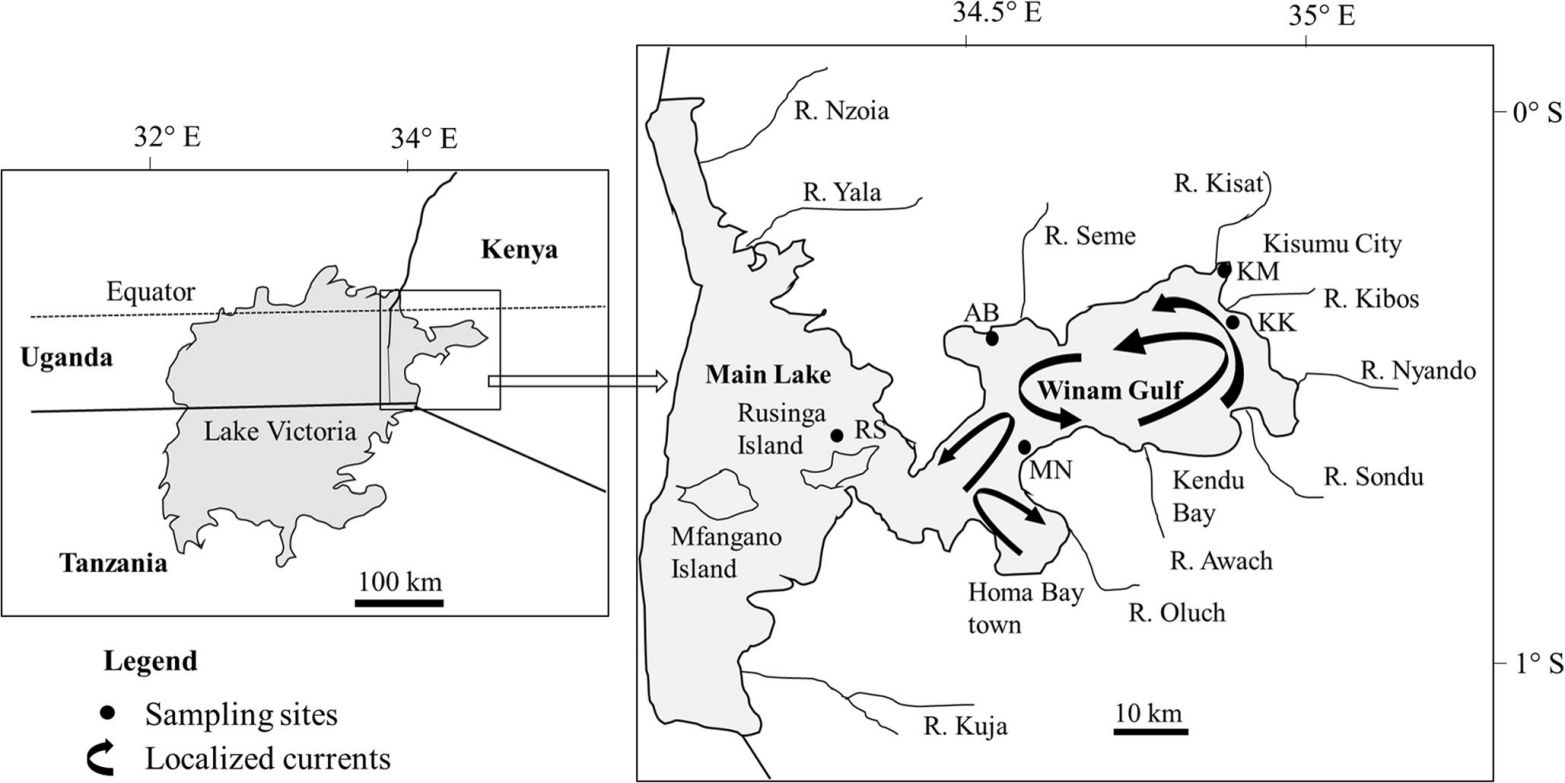
Map of Lake Victoria indicating the study area and sampling sites. Modified from Njuru et al. (2013); black arrows indicate localized currents based on Okely et al. (2010). AB, Asembo Bay (0° 11′ 10.2″ S 34° 23′ 35.8″ E); KM, Kisumu City (0° 05′ 16.4″ S 34° 44′ 59.0″ E); KK, Kisumu City outskirt (0° 09′ 41.4″ S 34° 44′ 51.6″ E); MN, Mainuga (0° 20′ 48.7″ S 34° 29′ 09.1″ E); and RS, Rusinga Island (0° 23′ 20.5″ S 34° 11′ 48.9″ E)

Lake Victoria, shared by Kenya, Tanzania and Uganda, is the world’s largest tropical lake and the second largest freshwater lake in the world, covering a total of 69,000 km^2^ with a mean depth of 40 m and maximum depth of 79 m (Okungu et al. 2005). It is located along the equator between 0.5° N and 2.5° S and 32° E and 34° E at an elevation of 1134 m above sea level, with a large catchment of 195,000 km^2^. The main river inlet (Kagera) drains through Rwanda, Burundi, Tanzania and Uganda, while the main river outlet is the Nile (Odada et al. 2004). The Kenyan portion of Lake Victoria lies just south of the equator between 0° 6′ S to 0° 32′ S and 34° 13′ E to 34° 52′ E; although its share is only 6% of the lake, it covers an area of about 4200 km^2^, of which 1400 km^2^ comprises the Winam Gulf (Lung’ayia et al. 2001). The Winam Gulf of Kenya (Fig. 1) is a shallow basin with an inshore (mean 4 m, maximum 6 m depth) and offshore zone (mean 12 m, maximum 43 m depth) (Crul 1995). The gulf receives high river inflows from western Kenya highlands (Njuru and Hecky 2005).

The equatorial climate at Lake Victoria basin is characterized by temperatures ranging between 20 and 35 °C and a mean annual rainfall between 1000 and 1500 mm (Okungu et al. 2005). A number of records (Crul 1995; Okungu et al. 2005; Yang et al. 2015) show that there are two main rainy seasons in the Lake Victoria basin: the long rains during March, April and May, resulting from the northward progression of the intertropical convergence zone (ITCZ), and the short rains in October and November, as the ITCZ moves southwards. The short rains can be intermittent or even fail in certain years (Crul 1995).

The five selected sites in the Kenyan part of Lake Victoria, both in the Winam Gulf and part of the main lake, are indicated in Fig. 1. The sampling sites—Asembo Bay (AB), Kisumu City (KM), Kisumu City outskirt (KK), Mainuga (MN) and Rusinga Island (RS)—were selected based on the perceived different types and levels of anthropogenic pressures. Also taken into consideration were the water currents, which influence the movement of contaminants in the gulf. Njuru et al. (2013) reported that contaminants entering the gulf through river inflows and municipal sources are largely retained, with minimal exchange into the main lake. The Rusinga Island sampling point (RS) is part of the main lake, buffered by the large water body, and was therefore regarded to represent the general characteristics of the main lake. As noted by Awange and Obera (2007) and Kundu et al. (2017), the KM site presents a part of the lake that suffers from the industrial and municipal wastewater discharges associated with urbanization. The Kisat River flows into the lake at Kisumu City, whereas the Kibos River feeds the lake at the outskirts of the city (Fig. 1). The site at the outskirts of Kisumu City (KK) is situated near the Kibos River inlet and is buffered by a large wetland area dominated by papyrus *Cyperus papyrus* L. The AB site, located on the northern shoreline, and the MN site to the south, are relatively rural sites within the mid-region of Winam Gulf. Rural farmlands in this region are potential sources of fertilizer runoff and sediment transport into the lake (Lung’ayia et al. 2001). Okely et al. (2010) showed limited horizontal mixing of water in these regions of Winam Gulf (AB, KM, KK and MN) (Fig. 1). The five sampling sites were therefore regarded to be potentially dissimilar in their physicochemical characteristics.

### Sampling and sample preparation

Sampling in the lake took place over 11 months from September 2016 to July 2017. Temperature, pH and electrical conductivity (EC) were measured monthly at approximately 10 cm below the water surface using portable VOLTCRAFT® meters (model PHT-02 ATC and LWT-02 ATC, Lindenweg, Germany). Water samples were taken at the same points and were filtered through 0.2-μm Acrodisc® PSF filters (PP membrane with a multilayer glassfibre pre-filter, PALL, Ann Arbor, USA) rinsed with 10 mL of distilled water prior to use, into 15-ml acid-rinsed polypropylene (PP) falcon tubes. They were then acidified to pH ≈ 2 with ultrapure acid (TraceSELECT® by Fluka, Steinheim, Germany). One filtered sample was acidified with HNO_3_ for analysis of major and trace elements and another one with HCl to determine dissolved nitrogen (DN) and dissolved organic carbon (DOC).

Surface sediments were taken monthly by scooping a 50-mL PP tube into the sediment or with a plastic corer (5 cm diameter). At each site, samples were taken in triplicate approximately 50 m apart. They were transported under cooled conditions and protected from light to Maseno University laboratory in Kisumu City and frozen at − 21 °C until further processing. Sediment core samples were taken at least twice at each of the sampling sites. Samples were transported within the corer to the laboratory and stored at − 21 °C until further processing. After thawing them for a short time under warm water, the cores were slipped out of the corer and sliced into layers of about 3 cm using a ceramic knife at three separate depths (‘0 cm’, ‘15 cm’, ‘25 cm’) and transferred into 50-mL PP falcon tubes. For further preparation, all sediment samples were oven dried at 90 °C until weight constancy was reached (2–3 days). Dried sediment samples were homogenized in a porcelain mortar and pestle, sieved through a 1-mm stainless-steel sieve and filled into 15-mL tubes for further storage.

Plants were handpicked wearing nitrile gloves, placed into 2-L plastic containers and covered with lake water for transport. During the study period, *V*. *cuspidata* samples were taken twice (October 2016 and May 2017) from the five sampling sites. *C*. *demersum* was found only at AB and RS in October 2016. Thereafter (from December 2016 to May 2017), *C*. *demersum* was not observed, coinciding with a period when most of inshore areas of the gulf were covered by *E*. *crassipes*. In the laboratory, samples were rinsed in Milli-Q® water. For *C. demersum*, a portion of the whole plant (approximately 5 g wet weight) was transferred into 50-mL tubes. For *V. cuspidata*, portions of stems and roots (approximately 5 g each) were separately put into 50-mL tubes. All plant samples were oven dried for 48 h to constant weight at 90 °C. The samples were shipped to the University of Vienna, Institute of Inorganic Chemistry laboratory, for further processing and chemical analysis.

Approximately 0.5 g of oven-dried, sieved sediment samples were weighed using an analytical balance into glass tubes, and 9 mL of 34% HNO_3_ (TraceSELECT® Fluka) and 1 mL of 30% H_2_O_2_ were added. Covered with air coolers, samples were processed for 2 h at 130 °C in a heating block. The leached samples were transferred to 20-mL volumetric flasks and topped up to volume using Milli-Q® water. Before analyses, samples were filtered through 0.2-μm PTFE membrane syringe filters (VWR, Radnor, USA). Reference samples comprising 0.2 g (dry weight) of marine sediment PACS-2 obtained from the National Research Council Canada (NRCC, Ottawa, Canada) were digested and diluted in the same manner as described above. A separate set of 0.5 g of the sediment samples was weighed into porcelain crucibles and ashed in a muffle furnace at 550 °C for 2 h to determine organic matter (OM) as percent ash free dry weight (AFDW) by loss of ignition (Wetzel and Likens 1991).

Plant tissue samples were homogenized with a mortar and pestle, and approximately 0.2 g was digested in 9 mL 34% HNO_3_ (TraceSELECT® Fluka) and 1 mL of 30% H_2_O_2_ using a microwave MARS XPRESS system (CEM Corporation, Smith Farm Rd, USA). The digested plant samples were transferred into 15-mL volumetric flasks and brought up to volume using Milli-Q® water. The leachates were filtered through 0.2-μm PTFE pre-syringe filters (VWR).

### Sample analyses

Water analyses for Na, Mg, K, Ca, Cr, Mn, Fe, Ni, Cu, Zn, As, Sr, Ag, Cd and Pb concentrations were carried out using total x-ray reflection fluorescence spectrometry (S2 PicoFox TXRF, Bruker; Billerica, USA), flame atomic absorption spectrometry (flame-AAS; Analyst 200 by PerkinElmer, Waltham, USA) or graphite furnace atomic absorptions spectrometry (GF-AAS) using a PinAAcle 900Z (Pelkin Elmer), depending on the concentrations of the elements and the LODs of the instrument used. DOC and DN were measured in the filtered acidified water samples using a TOC-VCPH total organic carbon analyser by Shimadzu, Kyoto, Japan. Trace element (Cr, Ni, Cu, Zn, As, Sr, Ag, Cd and Pb) concentrations in sediment and plant tissue were determined using total x-ray reflection fluorescence spectrometry (S2 PicoFox TXRF, Bruker) and, when necessary, graphite furnace atomic absorption spectrometry (GF-AAS) using a PinAAcle 900Z (Pelkin Elmer). Recovery rates from the analyses of reference samples as well as limits of detection (LODs) for sediment and plant samples are given in SM Table 1 and 2.

### Statistical analysis

Statistical analysis was done using IBM SPSS version 21. The Shapiro-Wilk test, which is very sensitive in detecting deviations from normality (Mohd and Yap 2011), was used to check for the normality of data distribution. The differences in spatial distribution of major and trace elements in water and sediment were determined by comparing site-specific means. Parametric one-way ANOVA was applied for comparison of means where appropriate. For the post hoc test, Tukey’s HSD was applied for pairwise mean comparisons where homogeneity of variance was established, whereas the Games Howell test was applied for unequal sample sizes and variances (Games and Howell 1976; Kim 2015). The non-parametric Kruskal-Wallis and Mann-Whitney *U* tests (for pairwise comparisons) were applied for data sets without normal distribution. All differences were tested for significance at *P* < 0.05.

The bioconcentration factors (BCFs) were determined according to the formula:

$$ {BCF}_{\left(\frac{x}{y}\right)}=\frac{Concentration\ of\ element\ in\ x}{Concentration\ of\ element\ in\ y} $$

in which the variables *x* and *y* stand for matrices that are compared with each other, such as water, sediment and macrophyte tissue.

### Evaluation of sediment quality

To evaluate the sediment quality, parameters that provide a reliable basis for classifying sediment as toxic or nontoxic were used following MacDonald et al. (2000), de Deckere et al. (2011) and Simpson et al. (2013). These parameters were threshold effect concentrations (TEC), the consensus-based levels below which toxic effects are rarely observed in sediment-dwelling organisms; consensus-based probable effect level (PEL), the concentrations above which toxic effects are generally observed; and severe effect level (SEL) at which sediments are considered heavily polluted and adverse effects occur in most benthic organisms. The trace element contents of all sediment samples, including the mean values for each sampling site, were compared with the respective metal guidelines.

## Results and discussion

### Water-dissolved components

**Fig. 2:**
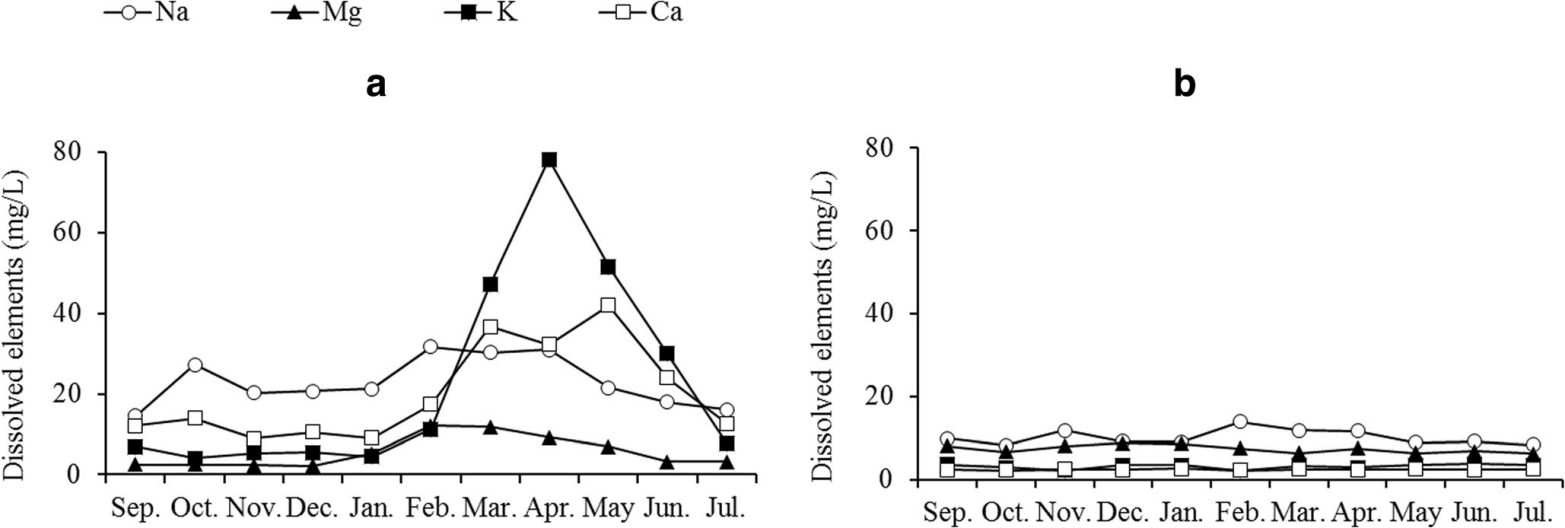
Temporal variation of major elements in surface water at Kisumu City (**a**) and Rusinga Island (**b**) sampling sites

The results for the physicochemical parameters, dissolved major and trace elements, as well as dissolved organic carbon (DOC) and dissolved bound nitrogen (DN) are presented in Table 1. The dissolved components were highest in Kisumu City (KM) and comparatively low at Rusinga Island (RS). Manganese was below the limit of detection (LOD) of 0.05 mg/L in nearly all the samples except for the KM site, where 1.36 to 2.18 mg/L was recorded between February and May. Ag and Cd were below detection limits in all samples (LOD 0.1 and 0.07 μg/L, respectively). Temporal trends (Fig. 2) indicate high levels of dissolved elements between March and May, especially at KM where for instance K increased from 11 mg/L in February to 78 mg/L in April; these results were mirrored in the electrical conductivity (EC) values. In the other four sampling sites, temporal variation was not prominent, which is reflected in the smaller standard deviations of the mean values at those sites (Table 1).

**Table 1:** Physicochemical parameters and dissolved major and trace elements (mean ± SD) of water in the study sites. Italic text: site with the highest mean concentrations for each parameter. Mean values of each parameter followed by same superscript letters do not differ significantly (*P* > 0.05)

Parameter	AB (*n* = 10)	KM (*n* = 11)	KK (*n* = 9)	MN (*n* = 8)	RS (*n* = 11)	EU/OECD guidelines
T (°C)	27.7 ^a^ ± 1.6	26.8 ^a^ ± 1.8	28.1 ^a^ ± 2.8	26.9 ^a^ ± 1.1	27.3 ^a^ ± 1.2	
pH	7.4 ^a^ ± 0.4	7.7 ^ab^ ± 1.2	6.9 ^a^ ± 0.5	7.9 ^b^ ± 0.4	*8.1*^*bc*^*± 0.7*	6.5–8.5
EC (μS/cm)	139 ^a^ ± 16	*272*^*b*^*± 158*	117 ^ac^ ± 29	142 ^a^ ± 13	93.1 ^c^ ± 15	1000
DOC (mg/L)	9.91 ^a^ ± 2.6	*17.4*^*a*^*± 7.9*	10.8 ^ab^ ± 4.6	9.67 ^a^ ± 2.0	4.99 ^b^ ± 2.1	
DN (mg/L)	0.531 ^ab^ ± 0.21	*1.32*^*b*^*± 1.1*	0.410 ^ac^ ± 0.11	0.610 ^bc^ ± 0.21	0.350 ^d^ ± 0.21	1.5
Na (mg/L)	17.4 ^a^ ± 2.2	*22.9*^*a*^*± 6.1*	19.6 ^a^ ± 4.4	20.2 ^a^ ± 2.5	10.6 ^b^ ± 3.1	
K (mg/L)	4.83 ^a^ ± 1.2	*23.1*^*b*^*± 25*	4.58 ^ac^ ± 2.0	4.82 ^a^ ± 1.0	3.11 ^c^ ± 0.5	
Mg (mg/L)	2.76 ^a^ ± 1.3	*5.57*^*a*^*± 3.8*	3.10 ^a^ ± 0.71	3.32 ^a^ ± 0.61	2.37 ^b^ ± 0.11	
Ca (mg/L)	10.3 ^a^ ± 1.3	*20.1*^*b*^*± 12*	12.7 ^ab^ ± 2.6	11.3 ^ab^ ± 2.1	7.3 ^c^ ± 0.90	
Cr (μg/L)	0.27 ^a^ ± 0.3	0.31 ^a^ ± 0.4	0.21 ^ab^ ± 0.2	*0.39*^*a*^*± 0.5*	0.14 ^b^ ± 0.1	50
Fe (μg/L)	450 ^a^ ± 500	*1110*^*b*^*± 900*	610 ^ab^ ± 600	710 ^ab^ ± 600	270 ^a^ ± 200	200
Ni (μg/L)	< 0.8	0.941 ^a^ ± 0.51	< 0.8	*2.21*^*b*^*± 0.72*	1.46 ^c^ ± 0.49	8.00
Cu (μg/L)	1.62 ^ab^ ± 0.7	1.93 ^ab^ ± 0.9	0.94 ^a^ ± 0.6	*2.18*^*b*^*± 0.8*	1.17 ^ab^ ± 1.0	20.0
Zn (μg/L)	130 ^a^ ± 60	190 ^ab^ ± 300	150 ^a^ ± 100	*200*^*ab*^*± 200*	50 ^b^ ± 30	70.0
Sr (μg/L)	120 ^a^ ± 10	*220*^*ab*^*± 130*	140 ^ab^ ± 20	140 ^b^ ± 20	90 ^c^ ± 10	
As (μg/L)	1.74 ^ab^ ± 0.3	*1.89*^*a*^*± 1.3*	1.69 ^ab^ ± 0.1	1.83 ^ab^ ± 0.4	1.47 ^b^ ± 0.3	50
Pb (μg/L)	1.09 ^ab^ ± 0.5	*1.21*^*ab*^*± 0.4*	1.01 ^ab^ ± 0.2	1.11 ^b^ ± 0.3	0.861 ^a^ ± 0.2	2.50

EU (2001), European Union; OECD (2007), Organisation for Economic Co-operation and Development: water quality guidelines for surface inland freshwater bodies

The comparatively high mean values of dissolved components at KM, which showed temporal variation, can be attributed to anthropogenic influences of the city. The KM site is located at an inshore part of Winam Gulf and is polluted by partially treated municipal and industrial discharges. Other sources of pollution include the washing of motor vehicles along the lakeshores and urban runoff from busy traffic and open garages, from where oil spills and particles of scrapped paint are washed into the lake. Moreover, several open domestic waste dump sites of Kisumu City are situated along the lake’s shore (Awange and Obera (2007), Kundu et al. (2017)). The role played by the Kisat River in polluting the lake at this site is equally important. In assessing the ecological integrity of the Kisat River, Kobingi et al. (2009) noted that the river drains through farmlands in its middle reach and becomes heavily contaminated by industrial discharge and poorly treated sewage from Kisumu City at the lower reach. In contrast, the RS site, which is buffered by the main water body of the lake, displays significantly lower mean values of EC and dissolved components (DN, Na, Mg, Ca, Sr), with significantly lower variations compared with the other sites. This agrees with the findings of Ochieng et al. (2006), who noted that seasonal variability is not significant in large lakes like Lake Victoria. At the KM site, the elevated levels of EC, major and trace elements in surface water coincided with the long rain season (March–May). This indicates that storm water surface runoff through the city, and the Kisat River, played a major role in the physico-chemical characteristics of water here. Although KK is located just at the outskirts of Kisumu City, and near the discharge point of the Kibos River, most of the water quality parameters were very distinct from KM but comparable with those of the main lake site (RS). This can be explained by two factors. The first is the buffering effect of the wetland through which water from the Kibos River enters the lake. A dense, approx. 3-km-wide belt of *C. papyrus* acts as a buffer and filter for incoming components. Numerous studies have shown that wetland plants can filter runoff water by increasing sedimentation and the uptake of contaminants, thereby protecting adjoining aquatic ecosystems (Verhoeven et al. 2006). Secondly, as shown by Okely et al. (2010) and indicated in Fig. 1, the waterbody at the KK site is rather isolated from the KM site by lake currents: waterborne materials dispersed from Kisumu City site are transported along a narrow stream of currents near the northern shoreline. Accordingly, materials entering the lake at KM will have limited horizontal mixing with water at KK. The mean concentrations of dissolved components at AB and MN sites were comparable, but higher than at RS, underlining other sources within the gulf, apart from Kisumu City. Runoff from rural farmlands adjacent to the lake could be a contributing source of contaminants (Lung’ayia et al. 2001). The high level of dissolved Ni, Cu and Zn at MN is also mirrored by the elevated levels in the surface sediment (especially for Ni and Zn) (Table 2). We therefore assume a diffuse contamination of dissolved metals rather than a point source of the respective metals.

**Table 2:** Concentration (mean ± SD) of elements (mg/kg dry weight) and organic matter (OM) content (%) in surface sediment in the sampling sites as compared with literature data. Italic text: site with the highest mean concentrations for each parameter. Mean values followed by same superscript letters for each parameter do not differ significantly in the current study (*P* > 0.05)

Parameter	AB (*n* = 30)	KM (*n* = 33)	KK (*n* = 27)	MN (*n* = 24)	RS (*n* = 33)	TEC	Shale
OM	9.39 ^a^ ± 4.6	*9.51*^*a*^*± 5.5*	6.23 ^b^ ± 3.0	2.37 ^c^ ± 1.2	3.83 ^d^ ± 2.2		
Cr	27.9 ^a^ ± 19	*56.9*^*b*^*± 21*	41.8 ^c^ ± 17	26.7 ^a^ ± 13	28.7 ^a^ ± 21	43.5	42
Ni	15.9 ^a^ ± 9.9	15.9 ^a^ ± *7.1*	19.1 ^a^ ± 8.6	*29.0*^*b*^ ± 11	17.3 ^a^ ± 6.9	22.7	18
Cu	28.2 ^a^ ± 16	*60.1*^*b*^ ± *63*	29.4 ^a^ ± 14	23.7 ^a^ ± 11	46.9 ^b^ ± 35	35.8	45
Zn	81.6 ^a^ ± 39	*295*^*b*^ ± *229*	107 ^c^ ± 39	105 ^ac^ ± 39	94.1 ^ac^ ± 29	121	95
As	2.69 ^ad^ ± 5.4	4.98 ^b^ ± *4.1*	2.12 ^a^ ± 1.4	*6.19*^*c*^ ± 3.1	1.19 ^d^ ± 0.4	9.79	4.7
Sr	60.1 ^a^ ± 19	71.1 ^a^ ± *49*	117 ^b^ ± 129	*603*^*c*^ ± 193	426 ^d^ ± 148		300
Ag	0.038 ^a^ ± 0.02	*1.36*^*b*^ ± *1.7*	0.031 ^a^ ± 0.01	0.037 ^a^ ± 0.02	0.023 ^c^ ± 0.01	1.0	0.1
Cd	0.193 ^a^ ± 0.25	*0.556*^*b*^ ± *0.72*	0.091 ^c^ ± 0.05	0.294 ^b^ ± 0.06	0.119 ^ac^ ± 0.05	0.99	0.3
Pb	6.82 ^a^ ± 3.4	*195*^*b*^ ± *273*	15.4 ^c^ ± 5.8	27.9 ^d^ ± 10	13.3 ^c^ ± 7.5	35.8	20

TEC - Consensus-based threshold effect concentration: Macdonald et al. (2000); Simpson et al. (2013)

Shale – Earth’s crust geochemical average background value: Turekian and Wedepohl (1961); Alloway (2013)

DN and DOC also varied distinctly between the sites within Winam Gulf and the RS site. Contamination of the lake with DN from the catchment is reflected in the high levels of DN in the gulf: KM (1.32 ± 1.1 mg/L), MN (0.601 ± 0.23 mg/L) and AB (0.523 ± 0.19 mg/L) compared with RS (0.347 ± 0.17 mg/L). Nitrogen enrichment of Lake Victoria stems mainly from sewage effluents and river inputs that drain agricultural lands and discharges into the gulf (Nyilitya et al. 2016). The mean DOC values in the four gulf sites (9.67–17.4 mg/L) were much higher than at RS (4.99 mg/L). Sobek et al. (2007) reported a median value of 5.71 mg/L from 7514 lakes from six continents, which is comparable with our findings from the main Lake Victoria body (RS), but lower than the mean values in Winam Gulf. In lakes, DOC can be autochthonous and/or allochthonous (Sobek et al. 2007). The results of our study can be explained by two factors. First, wetland vegetation around the inshore bays and gulfs of Lake Victoria is dominated by a highly productive emergent C_4_ sedge (*C. papyrus*) which generates large quantities of organic carbon under the favourable conditions of high solar insolation and abundant water (Loiselle et al. 2010). Second, intensive land use in the catchment may act as a significant source of organic carbon (Njiru et al. 2008). With regard to the spatial distribution of DOC in Lake Victoria, Loiselle et al. (2010) observed a limited exchange of organic carbon between Winam Gulf and the main lake, where only one-third of the DOC released into the lake at the gulf reached the open water. This is mirrored in our finding that DOC at KM was at least three times higher than at RS. In Winam Gulf, however, the nuisance floating macrophyte—the water hyacinth *E*. *crassipes*—dominated the water surface and was present during our study; its role in DOC cycling should be investigated in future studies. DOC is crucial in the chemistry of freshwater because it complexes heavy metals, making them less bioavailable to aquatic organisms (Winch et al. 2002). The DOC association with Fe complexation in the water (Knocke et al. 1992) is mirrored in the spatial distribution of dissolved Fe in our study.

Despite the spatio-temporal variations in the concentrations of dissolved major and trace elements in Lake Victoria, most of these parameters were below the water quality guidelines for surface inland waters, e.g. (EU 2001; OECD 2007) as indicated in Table 1. The levels of dissolved Fe and Zn were, however, higher than the recommended guidelines.

### Trace elements in sediment

**Fig. 3:**
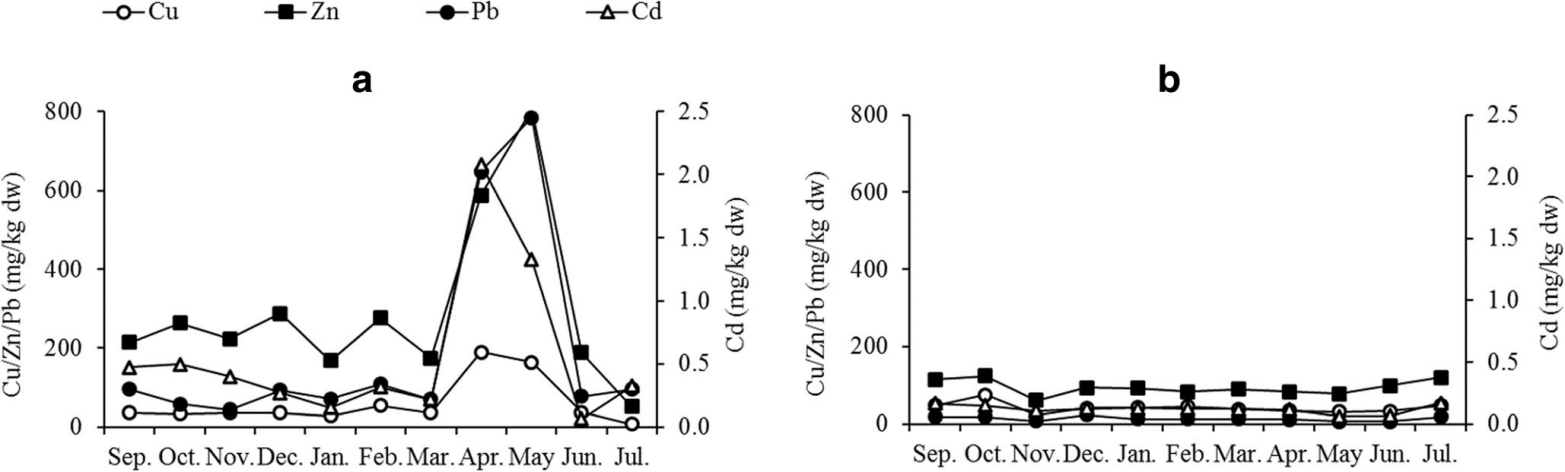
Temporal variation of selected heavy metals in surface sediment at Kisumu City (**a**) and Rusinga Island (**b**) sampling sites

Table 2 summarizes the concentrations of organic matter and trace elements in the surface sediment. The order of concentration of trace elements measured in the sediment was Sr > Zn > Pb > Cu > Cr > Ni > As > Ag > Cd. When interpreting the results, note that the digestion method used is considered to be ‘pseudo-total’ (Davidson 2013), so the actual concentration of the trace elements in sediment could be as much as 10–30% underestimated. The highest mean values for Cr, Cu, Zn, Ag, Cd and Pb were recorded at the KM site, with MN having the highest mean value for Ni, As and Sr (*P* < 0.05). The temporal trends indicate a sharp increase in the levels of Cu (fivefold), Zn (threefold), Cd (ninefold) and Pb (ninefold) from March to April at the Kisumu City site (Fig. 3). Temporal variation was not prominent at the other four sampling sites, as reflected in the smaller standard deviations of the means compared with KM (Table 2).

As was the case with the increase of dissolved elements in water, the contamination of the surface sediment at KM due to the impact of Kisumu City is evidenced in the much higher mean values of trace elements, which showed temporal variation during the study. This is attributed to both point and diffuse sources of contamination (Awange and Obera 2007; Kobingi et al. 2009; Kundu et al. 2017). The spatial distinctions in trace element levels in the surface sediment probably reflect the limited exchange of water-borne materials between the study sites. Njuru et al. (2013) observed that contaminants entering the gulf through river inflows and municipal sources are largely retained there, with minimal exchange into the main lake. Moreover, Okely et al. (2010) showed that localized water currents limited the transport of contaminants within the gulf (Fig. 1). The elevated levels of trace elements in KM surface sediment coincided with the long rain season (March–May), indicating that storm water surface runoff through the city and input through the Kisat River played a major role in the sediment characteristics here. As indicated in supplementary material (SM) Table 3, Cu and Zn had the highest positive correlation with OM (*r*_s_ = 0.53 for Cu and 0.52 for Zn) and showed a significant high positive correlation with each other (*r*_s_ = 0.72). This indicates that they are probably accumulated simultaneously from runoff and wastewater inputs. Skwierawski and Sidoruk (2014) concluded that high correlations among metals in freshwater sediment point to shared pollution and sediment enrichment sources. Cd occurs in phosphate fertilizer to varying degrees and in agricultural soils where phosphate fertilizer is applied (Roberts 2014). Runoff from agricultural lands is therefore a potential source of Cd contamination in Lake Victoria. In contrast, a potential source of Pb contamination at Kisumu City is the use of lead-based paints, which are yet to be phased out in Kenya (Kessler 2014). Finally, Pb-based fuel was widely used in Kenya before being phased out in the last decade, and roadside soils in Kisumu City were contaminated by Pb (Makokha et al. 2008). Runoff from contaminated roadside soils and open garages (with Pb-based paint scrapings) are therefore potential sources of Pb contamination in Lake Victoria. Most of the trace elements showed significant positive correlations with the organic matter content of the sediment (SM Table 3). Cu, Zn and Ag showed medium positive correlations with OM, while Cr, As and Cd showed weak positive correlations. Ni and Pb showed no significant correlations with OM, while Sr is the only element that had a significant negative correlation with OM. Lake Victoria is eutrophic (Kundu et al. 2017) and, as noted by Otachi et al. (2014), nitrates play a role in reducing Sr-organic matter complexes; this can partly explain the negative correlation we found in our study.

**Fig. 4:**
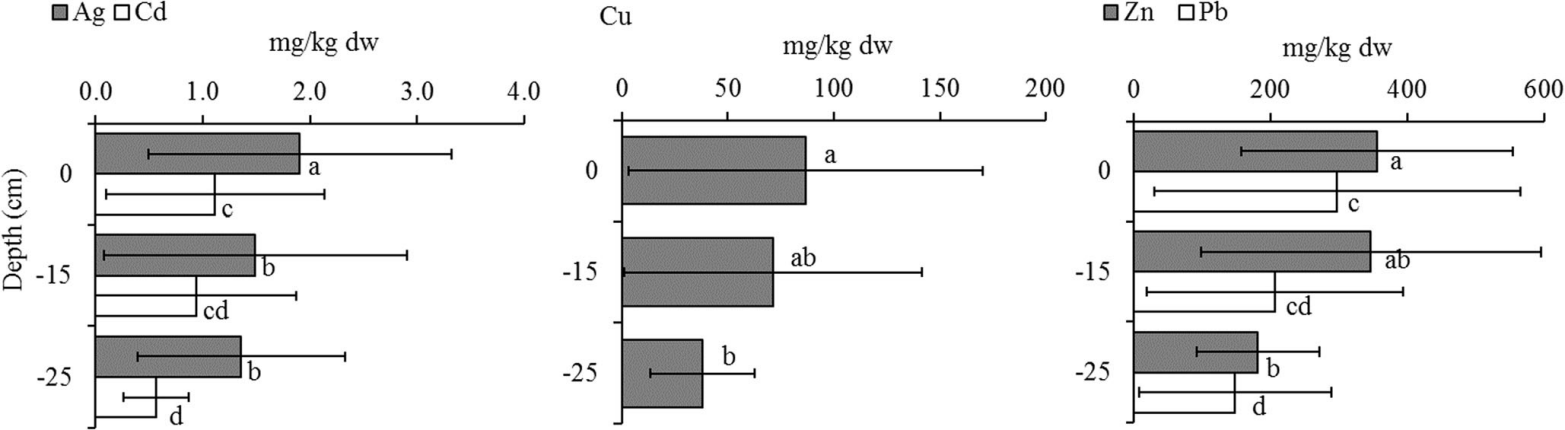
Concentrations of selected heavy metals in surface, − 15-cm and − 25-cm sediment layers at the Kisumu City sampling site. Error bars (mean ± SD) for each element followed by the same letters do not differ significantly (*P* > 0.05). For each sediment layer, *n* = 9

The distribution of trace elements showed no significant variation between the three sediment layers (surface ‘0 cm’, ‘− 15 cm’, ‘− 25 cm’) at the sampling sites, except for selected elements at the Kisumu City site. As shown in Fig. 4, the levels of Cu, Zn, Pb and Cd decreased gradually, with significantly higher concentrations in the surface layer than at − 25 cm (*P* < 0.05). The Ag concentration was higher in surface sediment than both at − 15 and − 25 cm (*P* < 0.05). Some studies have shown that anthropogenic pollutants, including heavy metals, have a vertical distribution pattern with higher levels in the top versus deeper layers. Skwierawski and Sidoruk (2014) found very high Cr, Zn and Pb concentrations in the uppermost sediment layer (0–10 cm) in the region of Plociduga reservoir (Poland), which received storm water, whereas Yang et al. (2010) reported that the greatest concentrations of Cu, Zn, Cd and Pb in an urban lake (China) were found in the surface sediment, with a rapid decrease between 0- and 15-cm layers, and little change in the 15–25-cm layers. The 0–10-cm layer of inshore sediment at Lake Victoria represents approx. 15 years (Ramlal et al. 2003). Accordingly, the surface sediment in our study (0–3 cm) has been deposited within the last 15 years. This implies that the input of Cu, Zn, Ag, Cd and Pb into the lake at Kisumu City has risen significantly over the past 15 years. Ag is of interest because there is very little information on its levels in Kenyan aquatic ecosystems. According to WHO (2002), emissions from smelting operations, manufacture and disposal of certain photographic and electrical supplies were among the major anthropogenic sources of silver in the biosphere. Recent studies such as McGillicuddy et al. (2017) and Yuan et al. (2018) have shown that the usage of silver nano-particles has increased in numerous consumer products such as textiles, medical products, domestic appliances, food containers, cosmetics, paints and nano-functionalised plastics in many parts of the world. These products release silver into aquatic ecosystems through wastewater discharge, which raises health and environmental concerns. This recent trend in the use of silver probably explains why Ag levels were elevated in surface sediment at the Kisumu City cite, where municipal wastewater effluent enters the lake.

Comparing the metal contents of surface sediment from this study with previous studies reveals that the heavy metal levels have continued to rise at KM, with at least a twofold increase in Cu (60.0 mg/kg), Zn (295 mg/kg) and Pb (195 mg/kg) within the last three decades. Onyari and Wandiga (1989) in the 1985 survey reported mean values for Cu (24. 8 mg/kg), Zn (144 mg/kg) and Pb (25.5 mg/kg), whereas Mwamburi and Oloo (1996) reported corresponding values of 30 mg/kg (Cu), 136.4 mg/kg (Zn) and 122.7 mg/kg (Pb). In Mwanza Gulf, Kishe-Machumu and Machiwa (2003) reported even lower values: Cu (21.6 mg/kg), Zn (36.4 mg/kg) and Pb (29.6 mg/kg). Ag levels have nearly doubled in the last 15 years since Ochieng et al. (2006) reported mean values 0.8 mg/kg at Kisumu City from samples taken in 2001, compared with 1.36 mg/kg from our study. Our results from the city outskirts (KK), the mid-gulf sites (AB and MN) and main lake (RS) are comparable with the findings from the previous studies.

Evaluating the sediment quality with various toxicity parameters highlights the degraded ecological status of the lake with regard to trace element contamination in the gulf. The mean values of Cr, Cu, Zn, Ag and Pb in the surface sediment at KM (Table 2) surpassed the TEC limits (MacDonald et al. 2000; Simpson et al. 2013). Further analysis indicates that 45.5% of the samples analysed for Cr (12–84 mg/kg) surpassed the PEL of 68 mg/kg (de Deckere et al. 2011), while for Cu (14–259 mg/kg), 15.2% of the samples surpassed the SEL of 110 mg/kg. For Zn (46–1093 mg/kg), 18.2% of the samples were above the PEL of 459 mg/kg (MacDonald et al. 2000). Pb ranged from 28 to 1188 mg/kg with a mean value of 195 mg/kg, which is higher than the SEL of 167 mg/kg (de Deckere et al. 2011). Note, however, that the mean values for all metals measured at the other four sampling sites were below the TEC limits, except for Ni at Mainuga (MN) and Cu at Rusinga Island (RS). 8.3% of the MN samples had Ni concentrations above the PEL of 48.6 mg/kg (MacDonald et al. 2000; de Deckere et al. 2011). These findings indicate that the sediment at MN is moderately polluted with Ni, while KM is moderately polluted with Cr, Zn, Ag and Cd and severely polluted with Cu and Pb. The mean values for arsenic, in contrast, were only minimally elevated in all sampling sites and were within the recommended sediment quality guidelines. This indicates that these values are probably the background levels and that there is currently no significant anthropogenic input of arsenic at the study sites.

### Trace element accumulation in macrophytes

#### *V. cuspidata*

**Fig. 5:**
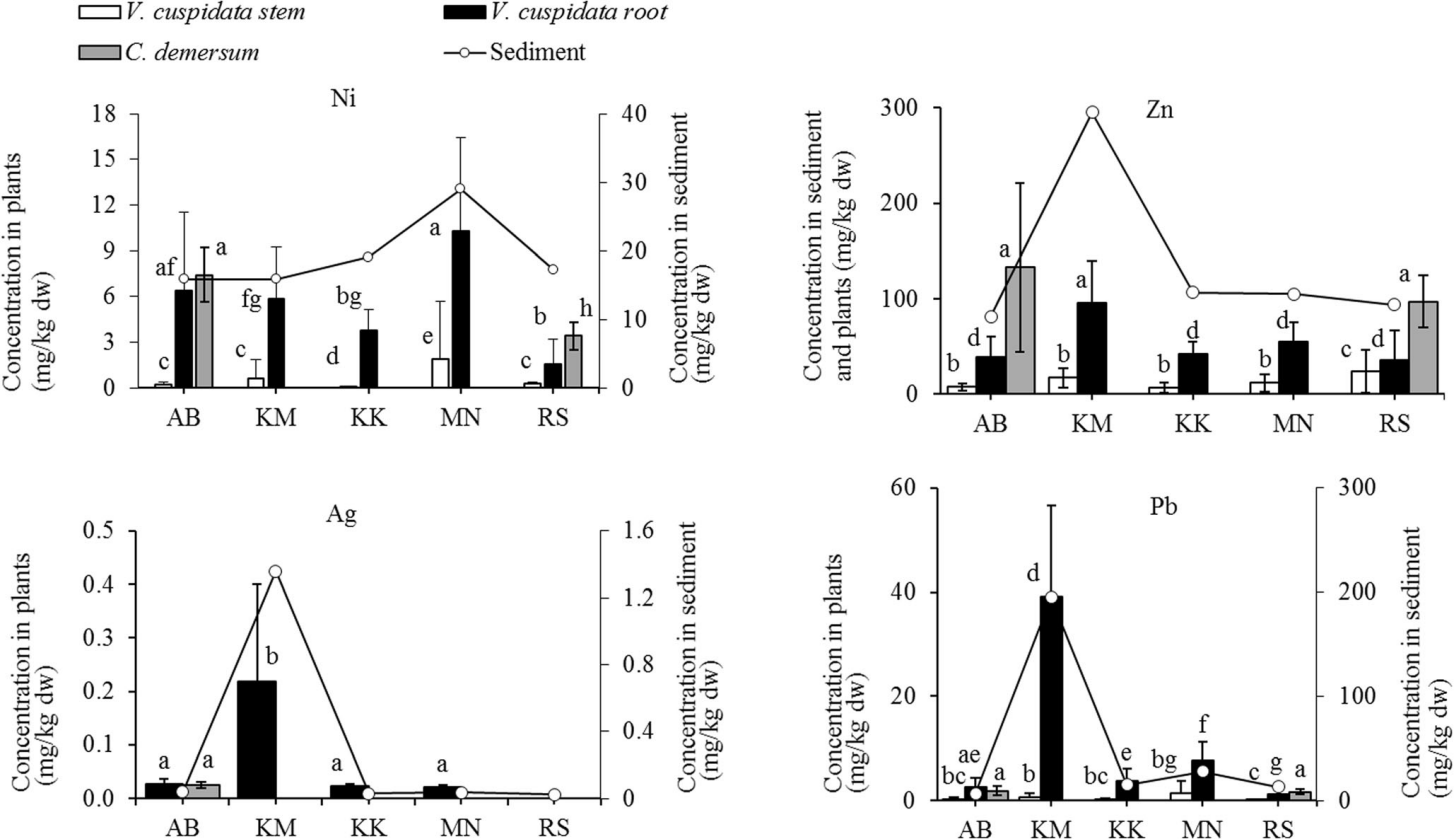
Concentration patterns of selected elements in *V. cuspidata* (stem and root) and *C. demersum* (whole plant) and the corresponding surface sediment content in different sampling sites. Surface sediment contents for Ni, Ag and Pb are indicated on the secondary axes. Error bars (mean ± SD) for elements in plant tissue followed by same letters do not differ significantly (*P* > 0.05). *C*. *demersum*: AB and RS (*n* = 10); *V*. *cuspidata*: AB and KM (*n* = 14); KK (*n* = 11): MN and RS (*n* = 12)

A total of 63 specimens of *V. cuspidata* were sampled: 14 each from AB and KM, 11 from KK and 12 each from MN and RS (Table 3). Ag was below the detection limit (0.015 mg/kg) in the stems at all sampling sites. Cd was also below the detection limit (0.005 mg/kg) in stem tissue except at MN. The roots and stems accumulated trace elements in the order: Zn > Sr > Cu > Pb > Cr > Ni > As > Cd > Ag. The concentrations were significantly higher in the roots than in the stems except for Sr at RS (*P* < 0.05). The spatial distribution of trace elements in the sediment is mirrored in the roots of *V. cuspidata*, with plants from the main lake site (RS) having the lowest concentrations of Cr, Ni, As, Sr, Ag, Cd and Pb compared with the four sites in Winam Gulf. Contamination of the lake at the Kisumu City site with Cu, Zn, Ag, Cd and Pb, and at the Mainuga site with Ni and Cd is well mirrored in the roots of *V. cuspidata*. Figure 5 depicts the correlations between plant and sediment concentrations for Ni, Zn, Ag and Pb; SM Figure 1 depicts the correlations for Cu and Cd. The BCF values for *V. cuspidata* stems and roots in relation to the sediment were below 1 for all elements (SM table 4).

**Table 3:** Concentration (mean ± SD) patterns of selected elements (mg/kg dw) in *V. cuspidata* (stem and root) and *C. demersum* (whole plant) in different study sites. Italic text: site and matrix with the highest mean concentrations for each element. Mean values followed by same superscript letters for each element do not differ significantly (*P* > 0.05)

Matrix	Site	*n*	Cr	Ni	Cu	Zn	As	Sr	Ag	Cd	Pb
*C. demersum*	AB	10	4.08^af^ ± 2.5	7.43^a^ ± 1.8	12.3^af^ ± 3.1	*133*^*a*^*± 88*	0.721^ah^ ± 0.40	133^a^ ± 29	0.025^a^ ± 0.01	0.198^a^ ± 0.06	1.92^a^ ± 0.9
	RS	10	2.53^a^ ± 1.2	3.42^b^ ± 0.92	6.47^b^ ± 1.8	97.3^a^ ± 28	0.342^abe^ ± 0.08	*138*^*a*^*± 14*	< 0.015	0.105^b^ ± 0.02	1.70^a^ ± 0.47
*V. cuspidata* stem	AB	14	0.812^bc^ ± 2.7	0.210^c^ ± 0.17	3.61^cd^ ± 9.6	7.66^b^ ± 3.7	0.241^bc^ ± 0.48	13.0^b^ ± 5.0	< 0.015	< 0.05	0.314^bc^ ± 0.39
	KM	14	0.502^bde^ ± 1.3	0.599^c^ ± 1.2	1.36^cd^ ± 1.7	17.1^b^ ± 11	0.120^c^ ± 0.04	11.8^b^ ± 7.5	< 0.015	< 0.05	0.628^b^ ± 0.88
	KK	11	0.064^c^ ± 0.03	0.104^d^ ± 0.04	0.634^c^ ± 0.40	6.92^b^ ± 5.1	0.188^c^ ± 0.02	5.62^c^ ± 1.5	< 0.015	< 0.05	0.261^bc^ ± 0.21
	MN	12	1.11d^e^ ± 2.5	1.88^e^ ± 3.8	2.72^d^ ± 3.8	11.1^b^ ± 8.7	0.360^c^ ± 0.41	10.3^b^ ± 3.5	< 0.015	0.030^ce^ ± 0.03	1.44b^g^ ± 2.41
	RS	12	0.187^e^ ± 0.13	0.263^c^ ± 0.13	7.49^be^ ± 7.2	23.8^c^ ± 23	0.223^c^ ± 0.05	10.4^b^ ± 8.4	< 0.015	< 0.05	0.187^c^ ± 0.13
*V. cuspidata* root	AB	14	*6.87*^*f*^*± 4.4*	6.39^af^ ± 5.2	11.8^ae^ ± 8.4	36.9^d^ ± 22	0.981^af^ ± 0.50	41.3^d^ ± 26	0.026^a^ ± 0.01	0.058^d^ ± 0.04	2.56^ae^ ± 1.8
	KM	14	6.44^f^ ± 2.9	5.88^fg^ ± 3.4	*18.3*^*f*^ ± *7.0*	96.2^a^ ± 44	*1.84*^*g*^*± 0.57*	52.6^e^ ± 17	*0.218*^*b*^*± 0.18*	0.153^a^ ± 0.17	*39.1*^*d*^*± 17*
	KK	11	5.56^f^ ± 4.4	3.77^bg^ ± 1.4	5.17^b^**±** 1.6	42.3^d^**±** 13	1.34^fg^ ± 0.33	32.9^d^ ± 6.9	0.022^a^ ± 0.004	0.035^cd^ ± 0.02	3.71^e^ ± 2.4
	MN	12	5.08^f^ ± 3.0	*10.3*^*a*^*± 6.2*	14.7^af^ ± 8.7	55.5^d^ ± 20	1.51^fgh^ ± 0.69	51.3^de^ ± 21	0.021^a^ ± 0.01	*0.252*^*a*^*± 0.21*	7.78^f^ ± 3.5
	RS	12	1.26^h^ ± 0.61	1.57^h^ ± 1.6	12.7^abf^ ± 11	35.6^d^ ± 31	0.517^a^ ± 0.35	13.1^b^ ± 10	< 0.015	0.011^e^ ± 0.01	1.16^g^ ± 0.86

The partitioning of trace elements, with higher accumulation in the roots than the stems, agrees with findings from other studies on emergent macrophytes (Brankovic et al. 2015; Galal et al. 2017). These plants take up and accumulate trace elements in the roots but restrict their translocation to the shoot and are regarded as excluders. Such metal excluders maintain a low shoot metal concentration over a wide range of substrate/soil concentrations (Baker 1981). This seems to be a protective measure. For instance, plants trap arsenate below ground to prevent access to above-ground reproductive tissues in order to prevent possible mutagenic consequences (Dhankher 2005).

Studies on trace element uptake and accumulation in *V. cuspidata* are scarce. Our findings differ considerably from those of Galal et al. (2017) on heavy metal accumulation in *V. cuspidata* from Ismailia Canal, Egypt. There, *V. cuspidata* roots accumulated higher levels of Ni (572–1059 mg/kg), Cu (452–1787 mg/kg), Zn (391–783 mg/kg), Cd (23.4–75. 8 mg/kg) and Pb (23.3–195 mg/kg) than in Lake Victoria: Ni (1.57–10.3 mg/kg), Cu (5.17–18.3 mg/kg), Zn (35.6–96.2 mg/kg), Cd (0.011–0.258 mg/kg) and Pb (1.16–39.1 mg/kg). Comparing the trace element contents of surface sediment in the two aquatic systems shows that, except for Cu and Cd, Lake Victoria had higher levels of most trace elements than Ismailia Canal. Since the respective concentrations in the sediments of the two systems cannot explain the highly contrasting findings on the concentrations in *V. cuspidata*, further investigations are warranted. As no previous studies on arsenic, Sr or Ag accumulation in *V. cuspidata* are available, only comparisons with other emergent macrophytes are possible. For instance, Favas et al. (2012) recorded mean arsenic concentrations of 0.90 mg/kg in the roots and rhizomes of *Typha latifolia* L. in Portugal, which is comparable with the range of means in our sampling sites (0.517–1.84 mg/kg). Yuan et al. (2018), in experimental treatments in the USA, recorded 4.49–8.87 mg/kg Ag in the root tissue of *Juncus effusus* L., which is much higher than our findings (0.02–0.54 mg/kg).

#### *C. demersum*

*C. demersum* was found only at the Asembo Bay (AB) and Rusinga Island sites (RS), and 10 specimens were taken from each site. The elemental concentrations are listed in Table 3. Ag was below detection limits in *C. demersum* at RS, but measurable in samples from AB. The plant accumulated the measured trace elements in the order: Sr > Zn > Cu > Ni > Cr > Pb > As > Cd > Ag. The plants at AB had higher concentrations of Ni, Cu, Ag and Cd than those at RS; however, except for Ag, those concentrations did not directly correspond to their respective levels in the water or sediment. The BCFs in relation to water were in the order: Cr > Ni > Cu > Pb > Zn > Sr > As (SM table 4). BCF was not calculated for Ag and Cd because they were below detection limits in water. Unlike *V. cuspidata*, which did not accumulate any of the trace elements higher than the levels in the sediment, *C. demersum* showed bioaccumulation: Zn (BCF = 1.03 at RS and 1.63 at AB), Sr (BCF = 2.21 at AB) and Cd (BCF = 1.03 at AB) in relation to the sediment. The concentrations (mean values) of Ni (5.43 mg/kg), Cu (9.39) and Pb (1.81 mg/kg) were much lower than in other studies. Kastratovic et al. (2014) recorded 11.2 mg/kg, 16.85 mg/kg and 7.48 mg/kg for the three elements in Skador, Montenegro, while the corresponding values in a eutrophic lake in China were 70 mg/kg, 32 mg/kg and 83 mg/kg (Xing et al. 2013). High accumulation of Sr (96.1–184 mg/kg) and a high BCF in relation to water (1276) in our study are higher than the findings of Kastratovic et al. (2014), who recorded a mean value of 22.9 mg/kg and BCF of 1.5.

*V. cuspidata* and *C*. *demersum* from the same sampling sites (AB and RS) showed a comparable accumulation of certain trace elements and a clear distinction in the accumulation of others. All stem concentrations except As were significantly higher in *C. demersum* than in *V. cuspidata* (*P* < 0.05). While the levels of Cr, Ni, Cu, As, Ag and Pb were not significantly different between *V. cuspidata* roots and *C. demersum* stems, the concentrations of Zn, Sr and Cd were significantly higher in the latter than in the former (*P* < 0.05). Macrophytes immersed in the water column have faster mass transfer rates, and a more poorly developed epidermal lipophilic cuticle, than emergent macrophytes. This means they have an enhanced uptake of metal(loid)s via their stems and leaves (Xing et al. 2010). Moreover, *C. demersum* is rootless, but the leaves are finely divided into a root like appearance, exposing a large surface area of the plant to water (high surface area to volume ratio) and thereby enhancing heavy metal uptake from the water column (Li et al. 2015). At the same time, the mobility and bioavailability of elements in the sediment can be very low, hence restricting uptake by emergent macrophytes (Lowry et al. 2012). As discussed above, *V. cuspidata*, like most emergent plants, restricts the acropetal transport of trace metals, explaining the very low levels these metals in their stems.

## Conclusion

The spatio-temporal distribution of metals in the water and surface sediment paints a picture of anthropogenic contamination in Winam Gulf. The impact of Kisumu City on the lake is evident by comparatively high mean values of dissolved components in the water and trace elements in the surface sediment, which showed temporal variation, with the highest values coinciding with the rainy season (March–May). Generally, the surface sediments in the gulf are moderately polluted with Cr, Ni, Cu, Zn, Ag and Pb, with severe Cu and Pb pollution occurring at the Kisumu City site. In addition, surface sediment (0–3 cm), representing sediment that has been deposited within the last 15 years, had significantly higher levels of Cu, Zn, Ag, Cd and Pb than deeper layers at KM, pointing to recent anthropogenic pollution.

Apart from Zn, Sr and Cd, which were higher in *C*. *demersum*, the concentrations of most elements were comparable between *C*. *demersum* stems and the roots of *V*. *cuspidata*. The spatial distribution of trace elements in the water and sediment is best mirrored in the roots of *V. cuspidata*, which accumulated Cr, Ni, Cu, Zn, Ag, Cd and Pb corresponding with their levels in the water and sediment. *V. cuspidata* is considered an excluder because it has a poor ability to transport trace elements from the roots to the stem. Hence, using the shoots as fodder does not pose a significant risk to livestock. Nonetheless, considering that the concentrations of Cr, Ni, Cu, Zn, Ag and Pb in surface sediment surpassed the consensus-based threshold effect concentrations in Winam Gulf, their levels in the fauna of the lake, which serve as food for the local population, should be subject to in-depth investigations.

## Electronic supplementary material

ESM 1 (DOCX 64 kb)
