# Supplementary material for: Distribution of arsenic, silver, cadmium, lead and other trace elements in water, sediment and macrophytes in the Kenyan part of Lake Victoria: spatial, temporal and bioindicative aspects
Source: Environ Sci Pollut Res Int. 2019 Nov 20;27(2):1485–98. doi: 10.1007/s11356-019-06525-9 (PMC6994457; doi:10.1007/s11356-019-06525-9)
Supplement: Supplementary file 1 — (DOCX 64 kb) [file 11356_2019_6525_MOESM1_ESM.docx]

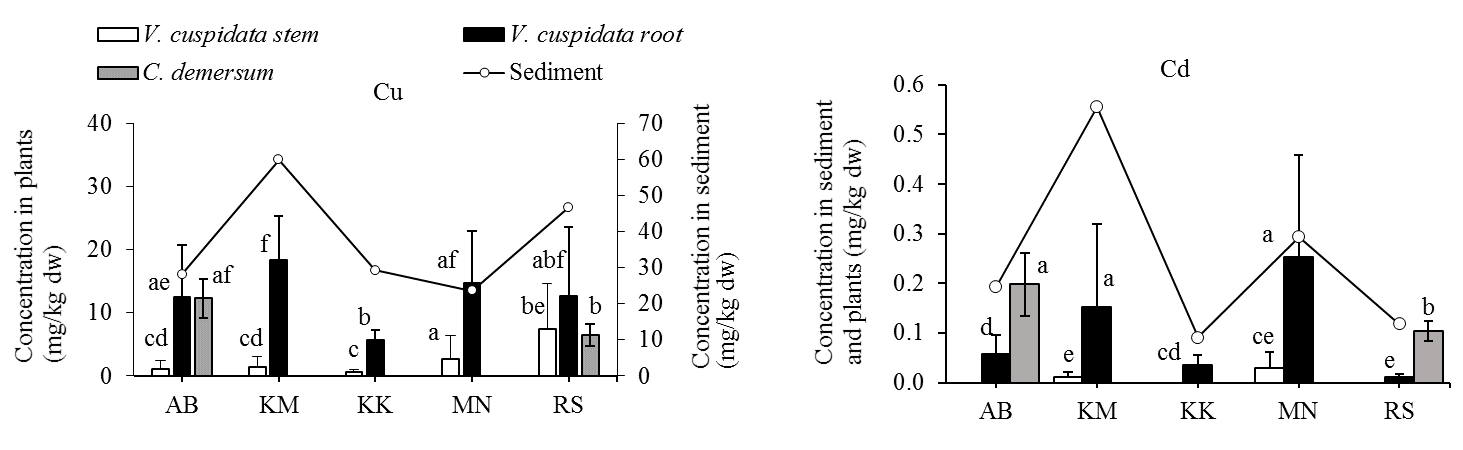


**SM Figure 1** Concentration patterns of Cu and Cd in *V. cuspidata* (stem and root) and *C. demersum* (whole plant) and the corresponding surface sediment content in different sampling sites. Surface sediment content for Cu is indicated on secondary axis. Error bars (Mean ± SD) for elements in plant tissue followed by same letters do not differ significantly (P > 0.05). *C*. *demersum* AB, *n* = 10; RS, *n* = 10: *V*. *cuspidata* AB, *n* = 14; KM, *n* = 14; KK, *n* = 11; MN, *n* = 12 and RS, *n* = 12.

**SM Table 1**: Recovery rates for the analyses of certified reference material PACS-2 by NRCC for respective methods and LODs under the applied conditions.

| element | method | reference value (mg/kg) | measured mean  (mg/kg) | recovery rate | LOD (mg/kg) |
| --- | --- | --- | --- | --- | --- |
| Cr | TXRF | 90.7 ± 4.6 | 94.8 ± 5.2 | 104.5 % | 0.4 |
| Ni | TXRF | 39.5 ± 2.3 | 35.6 ± | 90.0 % | 0.4 |
| Cu | TXRF | 310 ± 12 | 319 ± 21 | 102.9 % | 0.8 |
| Zn | TXRF | 364 ± 23 | 107.2 ± 5.5 | 101.8 % | 2.0 |
| As | TXRF | 26.2 ± 1.5 | 26.8 ± 2.0 | 102.3 % | 2.0 |
| Sr | TXRF | 276 ± 30 | 272 ± 32 | 98.6 % | 0.6 |
| Ag | GF-AAS | 1.22 ± 0.14 | 1.19 ± 0.28 | 99.5 % | 0.008 |
| Cd | GF-AAS | 2.11 ± 0.15 | 2.05 ± | 101.5 % | 0.003 |
| Pb | TXRF | 183 ± 8 | 182 ± 6 | 99.2 % | 2.0 |

**SM Table 2**: Limits of detection (LOD) for plant samples under applied conditions (all GF-AAS measurements except for Sr, which was measured with TXRF)

| Element | LOD (mg/kg) |
| --- | --- |
| Cr | 0.008 |
| Ni | 0.060 |
| Cu | 0.015 |
| Zn | 0.005 |
| As | 0.030 |
| Sr | 1.50 |
| Ag | 0.015 |
| Cd | 0.005 |
| Pb | 0.008 |

**SM Table 3:** Correlation values for OM and heavy metals in surface sediment (*P < 0.05; **P< 0.01).

|  | OM | Cr | Ni | Cu | Zn | As | Sr | Ag | Cd | Pb |
| --- | --- | --- | --- | --- | --- | --- | --- | --- | --- | --- |
| OM |  |  |  |  |  |  |  |  |  |  |
| Cr | 0.391** |  |  |  |  |  |  |  |  |  |
| Ni | 0.153 | 0.459** |  |  |  |  |  |  |  |  |
| Cu | 0.531** | 0.517** | 0.514** |  |  |  |  |  |  |  |
| Zn | 0.526** | 0.697** | 0.485** | 0.717** |  |  |  |  |  |  |
| As | 0.182* | 0.363** | 0.420** | 0.214** | 0.575** |  |  |  |  |  |
| Sr | -.332** | -0.102 | 0.520** | 0.273** | 0.097 | 0.189* |  |  |  |  |
| Ag | 0.506** | 0.569** | 0.258** | 0.320** | 0.665** | 0.619** | -.295** |  |  |  |
| Cd | 0.279** | 0.245** | 0.413** | 0.419** | 0.629** | 0.699** | 0.278** | 0.463** |  |  |
| Pb | 0.14 | 0.474** | 0.286** | 0.311** | 0.679** | 0.663** | 0.16 | 0.634** | 0.574** |  |

**SM Table 4:** BCF of trace elements in macrophytes

| Element | Site | *V. cupidata* Stem/ water | *V. cupidata* Stem/ Sediment | *V. cuspidata* root/ water | *V. cuspidata* root/ Sediment | *C. demersum*/ water | *C. demersum*/ sediment |
| --- | --- | --- | --- | --- | --- | --- | --- |
| Cr | AB | 3,009 | 0.029 | 25,444 | 0.25 | 15,351 | 0.15 |
|  | KM | 1,620 | 0.009 | 20,781 | 0.11 | ─ | ─ |
|  | KK | 304 | 0.002 | 26,481 | 0.13 | ─ | ─ |
|  | MN | 2,849 | 0.042 | 13,030 | 0.19 | ─ | ─ |
|  | RS | 1,336 | 0.007 | 9,031 | 0.04 | 18,097 | 0.09 |
|  |  |  |  |  |  |  |  |
| Fe | AB | 113 | 0.002 | 15,616 | 0.31 | 12,023 | 0.24 |
|  | KM | 193 | 0.004 | 16,728 | 0.38 | ─ | ─ |
|  | KK | 270 | 0.005 | 26,232 | 0.45 | ─ | ─ |
|  | MN | 206 | 0.004 | 13,764 | 0.26 | ─ | ─ |
|  | RS | 1,097 | 0.006 | 16,879 | 0.10 | 10,339 | 0.06 |
|  |  |  |  |  |  |  |  |
| Ni | AB | ─ | 0.013 | 7,983 | 0.40 | ─ | 0.47 |
|  | KM | 665 | 0.038 | 6,531 | 0.37 | ─ | ─ |
|  | KK | ─ | 0.005 | 4,718 | 0.20 | ─ | ─ |
|  | MN | 853 | 0.065 | 4,678 | 0.35 | ─ | ─ |
|  | RS | 176 | 0.015 | 1,044 | 0.09 | 2,279 | 0.20 |
|  |  |  |  |  |  |  |  |
|  |  |  |  |  |  |  |  |
| Cu | AB | 2,254 | 0.128 | 7,838 | 0.44 | 7,699 | 0.44 |
|  | KM | 714 | 0.023 | 9,641 | 0.31 | ─ | ─ |
|  | KK | 705 | 0.022 | 6,397 | 0.20 | ─ | ─ |
|  | MN | 1,234 | 0.115 | 6,699 | 0.62 | ─ | ─ |
|  | RS | 4,409 | 0.160 | 7,488 | 0.27 | 3,803 | 0.14 |
|  |  |  |  |  |  |  |  |
| Zn | AB | 60 | 60 | 308 | 0.48 | 1,038 | 1.63 |
|  | KM | 89 | 89 | 498 | 0.33 | ─ | ─ |
|  | KK | 46 | 46 | 282 | 0.40 | ─ | ─ |
|  | MN | 60 | 60 | 282 | 0.53 | ─ | ─ |
|  | RS | 467 | 467 | 699 | 0.38 | 1,909 | 1.03 |
|  |  |  |  |  |  |  |  |
| As | AB | 141 | 0.09 | 577 | 0.36 | 424 | 0.27 |
|  | KM | 63 | 0.02 | 969 | 0.36 | ─ | ─ |
|  | KK | 110 | 0.09 | 791 | 0.63 | ─ | ─ |
|  | MN | 200 | 0.06 | 839 | 0.24 | ─ | ─ |
|  | RS | 149 | 0.19 | 345 | 0.44 | 228 | 0.29 |
|  |  |  |  |  |  |  |  |
| Sr | AB | 104 | 0.216 | 333 | 0.69 | 1,069 | 2.21 |
|  | KM | 54 | 0.166 | 240 | 0.74 | ─ | ─ |
|  | KK | 41 | 0.048 | 240 | 0.28 | ─ | ─ |
|  | MN | 72 | 0.017 | 356 | 0.08 | ─ | ─ |
|  | RS | 119 | 0.026 | 151 | 0.03 | 1,483 | 0.32 |
|  |  |  |  |  |  |  |  |
| Ag | AB | ─ | ─ | 136 | 0.71 | ─ | 0.65 |
|  | KM | ─ | ─ | 1091 | 0.16 | ─ | ─ |
|  | KK | ─ | ─ | 112 | 0.72 | ─ | ─ |
|  | MN | ─ | ─ | 102 | 0.55 | ─ | ─ |
|  | RS | ─ | ─ | ─ | ─ | ─ | ─ |
|  |  |  |  |  |  |  |  |
| Cd | AB | ─ | ─ | 82 | 0.30 | ─ | 1.03 |
|  | KM | ─ | ─ | 218 | 0.27 | ─ | ─ |
|  | KK | ─ | 0.10 | 50 | 0.39 | ─ | ─ |
|  | MN | ─ | ─ | 360 | 0.86 | ─ | ─ |
|  | RS | ─ | ─ | 16 | 0.10 | ─ | 0.88 |
|  |  |  |  |  |  |  |  |
| Pb | AB | 286 | 0.046 | 2,326 | 0.38 | 1,743 | 0.28 |
|  | KM | 523 | 0.003 | 32,577 | 0.20 | ─ |  |
|  | KK | 261 | 0.017 | 3,708 | 0.24 | ─ |  |
|  | MN | 1,310 | 0.052 | 7,072 | 0.28 | ─ |  |
|  | RS | 207 | 0.014 | 1,294 | 0.09 | 1,885 | 0.13 |
